# Design of a Real-Time Salinity Detection System for Water Injection Wells Based on Fuzzy Control

**DOI:** 10.3390/s21093086

**Published:** 2021-04-28

**Authors:** Bo You, Yuandong Yue, Mingxiao Sun, Jiayu Li, Deli Jia

**Affiliations:** 1 Heilongjiang Provincial Key Laboratory of Complex Intelligent System and Integration, Harbin University of Science and Technology, Harbin 150080, China; sunmingxiao@hrbust.edu.cn (M.S.); 1920510074@stu.hrbust.edu.cn (J.L.); 2 School of Automation, Harbin University of Science and Technology, Harbin 150080, China; 1920510072@stu.hrbust.edu.cn; 3 Department of Oil & Gas Production Equipment, PetroChina Research Institute of Petroleum Exploration & Development, Beijing 100083, China; jiadeli422@petrochina.com.cn

**Keywords:** salinity, conductivity, integrator circuit, fuzzy control

## Abstract

Salinity is an important index of water quality in oilfield water injection engineering. To address the need for real-time measurement of salinity in water flooding solutions during oilfield water injection, a salinity measurement system that can withstand a high temperature environment was designed. In terms of the polarization and capacitance effects, the system uses an integrator circuit to collect information and fuzzy control to switch gears to expand the range. Experimental results show that the system can operate stably in a high-temperature environment, with an accuracy of 0.6% and an uncertainty of 0.2% in the measurement range of 1–10 g/L.

## 1. Introduction

In China, oil fields are complex and diverse, and most reservoirs are continental deposits. Reservoir heterogeneity is severe and natural energy is weak. Therefore, it is necessary to provide supplemental energy for future development. With the increasing demand for petroleum energy in various sectors of the society, the production of oilfield must be increased, which can lead to the depletion of oil resources that are easily accessible. Therefore, there is a constant increase in the depth of oil mining wells, and the exploration environment is becoming increasingly complex [1]. To make accessing deep deposits easier, scholars have proposed traditional water flooding technology, fine water injection technology, supercritical water flooding technology, and other water drive technologies [2,3]. Water flooding technology was previously developed, and this research is relatively mature. This method proposes injecting water into reservoirs during oilfield development and uses water as a medium to drive the reservoir to improve oil recovery. Owing to its advantages of low cost and high efficiency, it has become a commonly used method in petroleum production engineering for oil companies worldwide [4]. Currently, water drive technology remains the most economical production technology that is widely used in most oilfields in China.

Most water drive projects in China are conducted by the reinjection of oilfield wastewater. Oilfield wastewater reinjection saves numerous water resources. However, detecting and controlling the salinity of reinjected water after oil–water separation treatment is necessary. If water with high salinity is injected into the well, the ions in the solution may react with the ions in the formation, block the formation channel, destroy the formation structure, and cause irreparable damage to the original formation. If water with insufficient salinity is injected into the well, the wastewater treatment process will increase, thereby increasing the crude oil production cost [5].

To successfully manage the water injection and recovery processes, the salinity of the water solution in water injection wells should be detected in real time. Based on the differences in the volume mass, conductivity, refractive index, density, and sound velocity of solutions with different salinity conditions, the detection of salinity mainly includes a direct measurement method (i.e., the gravimetric method) and four indirect measurement methods (i.e., acoustic, density, light, and conductivity methods). The measurement of salinity using the gravimetric method uses hydrostatic weighing. Direct measurement of the mass of the solution per unit volume results in the highest accuracy for the salinity of the solution. However, it requires manual operation and has a long measurement period. Therefore, it does not meet the requirements of real-time detection. The acoustic method used for salinity measurement uses sonar technology. In liquids with different salinities, the sound velocities vary also. The salinity of the measured liquid can be obtained according to the return time of the ultrasonic wave. This method is more suitable for seawater salinity measurement. However, owing to its high cost and poor anti-interference ability, it is inappropriate for complex industrial environments.

The measurement of salinity using the density method requires a precision vibrating tube densimeter. A vibration tube made of magnetic material was connected to the object to be tested, and the electromagnetic driving coil and detection coil were installed beside the vibration tube. When the driving coil is supplied with a pulse exciting current, the vibrating tube vibrates under the magnetic force of the coil and a current with the same vibration frequency is generated in the detection coil. The liquid density in the tube was first calculated according to the vibration frequency of the vibration tube, followed by the salinity. Schmidt et al. [6] proposed a method for measuring seawater density using a vibrating flow densitometer. The uncertainty of the method is 2 × 10^−6^ in the range of 5–35 °C, which improved the measurement accuracy. However, owing to the required volume of the instrument, embedding other devices is difficult. 

Two classifications of sensors, fiber gratings and interferometers, are used as salinity sensors. In studies by Cong et al. [7] and Liu et al. [8], a salinity sensor with fiber grating was proposed, and its salinity sensitivity was 10.4 pm/%. Gentleman and Booksh [9] used a multimode optical fiber to measure the salinity of a liquid based on the principle of surface plasmon resonance. Compared with the traditional prism refractive index method, the sensitivity of the optical fiber increased to 200 pm/%. A salinity sensor based on photonic crystal fiber was proposed by Lee et al. [10], where the sensitivities of x-polarization and y-polarization are 5405 and 5675 nm/RIU, respectively. There are many types of sensors based on the interferometer method, such as the Fabry–Perot interferometer structure [11], rectangular optical microfiber Sagnac interferometer structure [12], C-type fiber structure [13], tapered structure [14], and single-mode multimode–single-mode (SMS) structure [15]. A high-sensitivity salinity sensor based on a coreless optical fiber was proposed by Lin et al. [16]. The sensitivity of the sensor reaches −3.42 nm/%, which is considered an appropriate sensitivity. In a study by Hussain et al. [17], two schemes for measuring the salinity of a liquid using smart phones were proposed, the feasibility was verified, and the portability of the salinity sensor was improved. Doulamis et al. [5] proposed a project called Waterspy in which a quantum cascade laser with the Ernie effect was used as the light source, and an optical fiber-coupled photodetector was used as the sensor for water quality detection. Its sensitivity can be used to detect pathogenic bacteria. Although measurement technology based on the light method has become increasingly popular, it is still not suitable for the measurement environment in water injection wells.

The salinity of a liquid can be measured based on its conductivity. The conductance method has the advantages of a clear principle, simple structure, easy implementation, low cost, fast response, and no fluid interference; therefore, it is widely used in industrial measurements [18,19,20]. According to the different working principles of sensors, conductivity sensors are divided into electromagnetic induction conductivity and electrode conductivity. Generally, the probe of an electromagnetic induction conductivity sensor comprises two metal coils: an excitation coil and an induction coil. An alternating current (AC) excitation signal was applied to the excitation coil of the sensor probe to induce an electric field. The induced electric field is transmitted to the induction coil through the measured solution, and an AC output voltage forms on the induction coil. The AC output voltage and conductivity of the measured solution form a monotonic nonlinear function, and the conductivity of the solution, can be obtained through calibration. In a study by Parra et al. [21], the electromagnetic induction conductivity sensor is applied to groundwater monitoring in a smart city with a measurement range of 0.58–73 mS/cm, and the maximum relative error is 8%. Tang et al. [22] designed a hardware circuit with capacitor reactance compensation based on the principle of capacitor reactance elimination. The maximum relative error was 3.58% in the measurement range of 0.2–30 mS/cm. The method based on electromagnetic induction conductivity sensors is a non-contact ranging method, which has the characteristics of high sensitivity and fast response, although it is vulnerable to electromagnetic interference. An electrode-type conductivity sensor is a form of contact measurement. The common method is to measure the conductivity of the solution in a conductivity cell composed of electrodes, and the measured conductivity of the solution is used in calculating salinity. The electrode conductivity sensor can be classified as two-electrode, three-electrode, four-electrode, and so on. Ramos et al. [23] designed a four-electrode conductivity sensor in which two electrodes were placed in a uniform electric field, and the other two electrodes were measured. This scheme effectively avoids the measurement errors caused by the polarization effect. The measurement range was from 0.05 to 5 S/m, the maximum error rate was 1.86%, and the uncertainty was 0.53%. The seawater salinity detection system designed by Huang et al. [24] integrates four electrodes into a measuring board with a conductivity accuracy of 0.03 mS/cm. The system detects the salinity once every 10 s and can run continuously for 30 days, thus reducing the volume and power consumption of the sensor. Kim et al. [25] designed a sensor module based on microfluidics. The flow control device of the module was composed of a metal film and a microfluidic control channel. Conductivity and temperature were measured simultaneously using microelectromechanical systems technology, and the mineralization degree was calculated. In the 5-day experimental data, the maximum average errors of the conductivity measurement and mineralization measurement were approximately 6% and approximately 8%, respectively. To optimize electrode materials, a capacitive coupling electrolytic conductivity sensor using a high-potassium barium strontium titanate material was proposed by Huck et al. [26]. Experiments verified that it could reduce the pollution on the electrode caused by continuous contact measurement and improve the life of the electrode. Chiang and Chang [27] designed a system to convert the degree of liquid mineralization into frequency detection. The system uses a pulse signal as the output, with a measurement range of 60–270 g/L and a measurement error of 2.57%. Joseph et al. [28] proposed the use of artificial diamond as an electrode material, which can accurately measure the conductivity by more than four orders of magnitude ($10-10^{5}\;\mu S/cm$) and can operate in extreme pH conditions (98% sulfuric acid). This significantly improves the stability of the conductivity sensor. Continuing the research of Chiang and Chang [27], Chiang et al. [29] optimized the process and reduced the measurement error by 0.47%.

Considering the requirement for real-time detection of salinity of water flooding solutions in oilfield wastewater reinjection and research on the aforementioned electrode conductivity sensors, we present a real-time detection system for the degree of mineralization, which is useful for undergrounding high-temperature environments. Based on the two-electrode conductivity measurement method, the voltage at both ends of the conductivity sensor and gear resistance was measured by an integrated circuit to reduce the error caused by polarity conversion. Fuzzy control is used in switching the gear resistance to different gears to increase the measurement range. The relationship model of conductivity, temperature, and salinity was obtained experimentally, and the temperature compensation and calibration of the system were performed. Finally, the accuracy, repeatability, and high-temperature resistance of the system were determined experimentally.

## 2. Polarization Effect, Capacitance Effect, and Methods of Reducing Error

**Figure 1:**
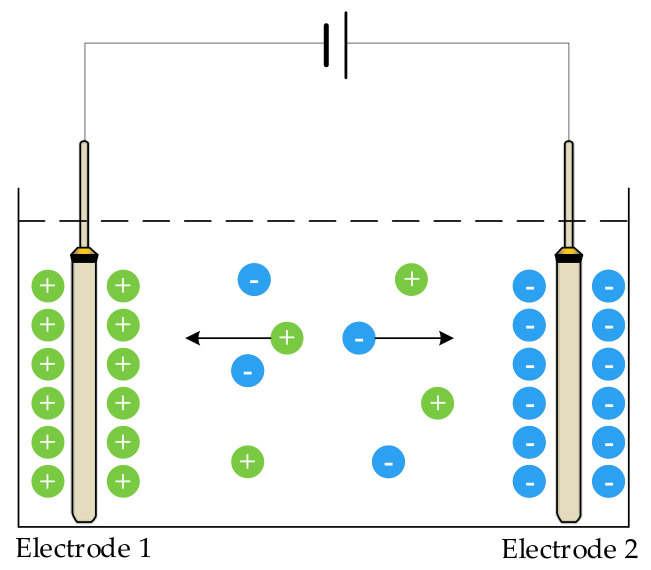
Schematic diagram of the polarization effect.

When a direct current (DC) voltage is applied to an electrode, ions in the solution move in the direction of the voltage; the positive ions move to the negative plate, and the negative ions move to the positive plate. As shown in Figure 1, electrodes 1 and 2 are two metal plates of the sensor, which are completely immersed in the solution to be measured. When a DC was applied to the two electrodes, the charged ions moved under the action of the electric field; that is, a polarization effect occurred at both ends of the electrode, resulting in a potential difference opposite to the original electromotive force, blocking the current flow, increasing the equivalent resistance of the solution, and causing measurement error.

**Figure 2:**
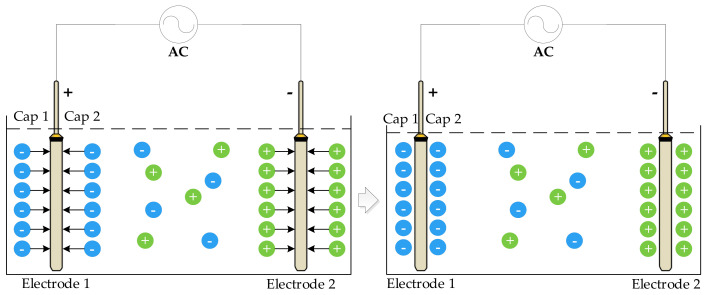
Schematic diagram of the capacitance effect.

To eliminate the influence of the polarization effect, an AC signal is typically used as the excitation source. When an AC signal is applied to the solution to be measured, double-layer capacitance is generated at both ends of the electrode, and an inter-electrode capacitance is generated between the two electrodes [30]. As shown in Figure 2, the two electrodes were completely immersed in the solution. When AC was applied to the two electrodes, voltage on electrodes 1 and 2 changed once every half cycle. When electrode 1 changed from positive to negative owing to the slow movement of conductive ions in the solution, the voltage between the electrodes was greater than the power supply voltage, resulting in a capacitance effect and reducing measurement error. Capacitance 1 and capacitance 2 in the figure are double-layer capacitors.

**Figure 3:**
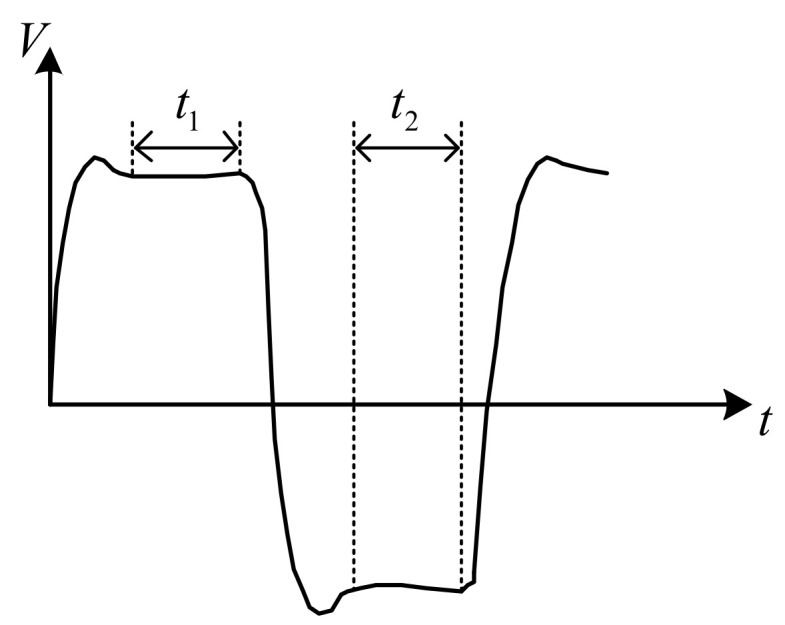
Waveforms at both ends of the sensor.

To further reduce the measurement error, a bipolar square-wave signal was used in the system. When the frequency is appropriate (in the case of a low-frequency excitation signal detecting a high conductivity solution or a high-frequency excitation signal detecting a low conductivity solution, it will produce an unacceptable measurement error, which is not appropriate), the square wave will experience a small period, equivalent to half the normal period, and the square wave experiences a period of little influence in a half cycle. When the polarity of the power supply changes, the capacitance effect of the electrode reaches its maximum value. The polarization effect of the electrode reaches its maximum just before the polarity of the power supply changes. During this period, the charge and discharge cycles of the equivalent capacitance were completed, and the polarization effect began. If the voltage signal is obtained during this period, a more accurate conductivity value is obtained, as shown in Figure 3.

Figure 3 shows how the influence of the capacitance and polarization effects of the electrode on the system can be effectively reduced by collecting the voltage at both ends of the conductivity sensor and the gear resistor during $t_{1}$ and $t_{2}$. The use of an AC signal to reduce the polarization effect of the electrode and DC signal to reduce the capacitance effect of the electrode can effectively improve the measurement accuracy.

The above analysis demonstrates that the AC signal must be input to the system to reduce the polarization effect of the electrode. To reduce the influence of the electrode capacitance effect, we need to measure the voltage signal, which is less affected by the polarization and capacitance effects. This requires that the signal acquisition circuit of the detection system be able to accurately collect the DC voltage in a very short time (millisecond level). In this study, an integrator circuit was used as the core circuit of the signal acquisition unit.

## 3. Hardware Circuit

**Figure 4:**
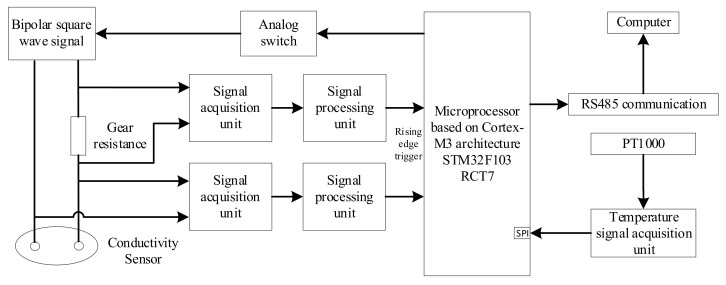
Overall structure of the system.

The hardware structure of the detection system is composed of a conductivity sensor and a hardware circuit. A conductivity sensor was used to detect the conductivity of the solution and converts the conductivity value into an electrical signal. The hardware circuit generated an excitation signal to excite the conductivity sensor and process the signal generated by the conductivity sensor. The overall architecture of the hardware system is shown in Figure 4.

The bipolar square wave signal, gear resistance, and conductivity sensor constitute a path. The microprocessor generated a bipolar square wave signal by controlling the analog switch. The bipolar square wave signal generated an AC voltage on the gear resistance and conductivity sensor. The signal acquisition unit collects the voltage on the two modules and transmits to the signal processing unit with the integrator circuit as the core. The signal processing unit converts the voltage into a time value and sends it to the microprocessor. The voltage at both ends of the gear resistance and conductivity sensor was calculated according to the discharge time of the integrator circuit, and the equivalent resistance value of the conductivity sensor was indirectly calculated to obtain the solution conductivity. The temperature measurement unit used a high-precision platinum thermal probe (PT1000, Hangzhou MEACON Automation Technology Co., Ltd., Hangzhou, China) as the temperature sensor, and the temperature value was displayed by the microprocessor through a software look-up table. Subsequently, the salinity value of the solution was transmitted to the upper computer through the RS485 communication module through temperature compensation.

### 3.1. Signal Acquisition Unit

**Figure 5:**
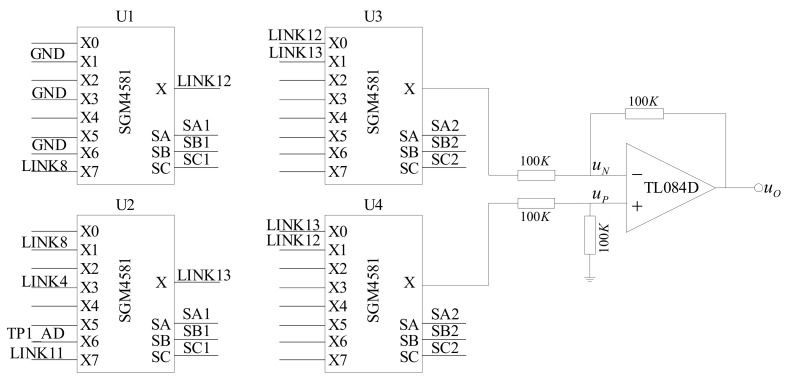
Circuit diagram of the signal acquisition unit.

According to the conductivity signal acquisition method proposed in this paper, the signal acquisition circuit must have the ability to collect the signal once every half of the working cycle. First, we must select the object to be detected, which determines whether the signal acquisition unit detects the conductivity or the temperature. Second, we need to convert the negative half-cycle signal into a positive signal and send it to the signal processing unit. A hardware circuit diagram of the information acquisition unit is shown in Figure 5.

U1, U2, U3, and U4 are analog switches in which the control pins SA, SB, and SC of U1 and U2 are connected in series with the microprocessor and controlled by the same group of control signals (SA1, SB1, and SC1). The control pins of U3 and U4 are connected in series and controlled by another group of control signals (SA2, SB2, and SC2). GND in U1 is a digital ground wire, LINK8 in U2 is connected with the inner electrode of the conductivity sensor, LINK4 is connected to +2.5 V, TP1_AD is connected with the temperature signal acquisition unit, U1 common output terminal is connected with X0 pin of U3 and X1 pin of U4, and common output terminal of U2 is connected with X1 pin of U3 and X0 pin of U4, respectively. Further, TL084 is an operational amplifier, and a subtractor is formed by four 100 KΩ resistances.

The control signals SA1, SB1, and SC1 determine the object of the signal acquisition. When X0 is ON, the voltage between the digital ground and the analog ground is collected. When X1 is ON, the voltage at both ends of the conductivity sensor is collected. When X7 is ON, the voltage at both ends of the gear resistance is collected. The X3 and X6 pins are connected to the temperature acquisition unit.

### 3.2. Signal Processing Unit

**Figure 6:**
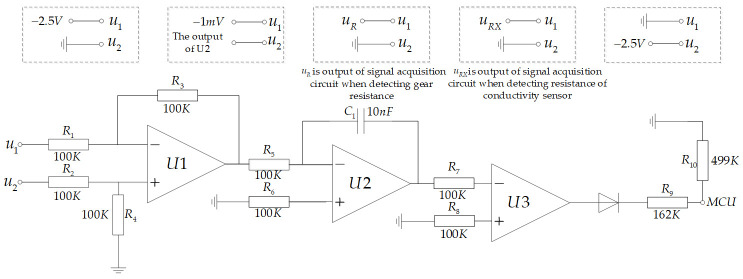
Schematic diagram of the measurement module.

The hardware circuit of the signal processing unit is shown in Figure 6. To measure the output voltage of the object to be detected using the characteristic that the output slope of the integrator circuit is only related to the input voltage.

The signal processing unit is primarily divided into three parts: the differential circuit, integrator circuit, and zero-crossing comparator parts. R_1_, R_2_, R_3_, R_4_, and U1 constitute the differential circuit; u_1_ and u_2_ are the two input terminals of the differential circuit, which are primarily used to collect the potential difference between them. Meanwhile, R_5_, R_6_, C_1_, and U2 constitute an integrator circuit, and R_7_, R_8_, and U3 constitute a zero-crossing comparator. The microprocessor collects the discharge time of the integrator circuit through a zero-crossing comparator to calculate the charging voltage.

**Figure 7:**
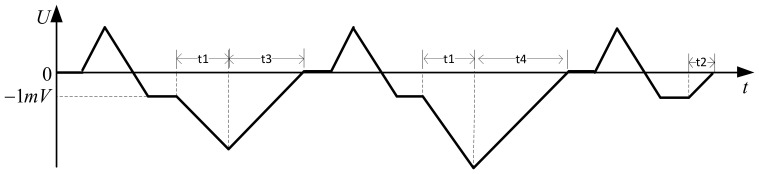
Voltage change curve of the U2 output.

The voltage measurement of the integrator circuit is primarily divided into two stages: reverse charging and discharging. For the measurement process, the voltage change curve of the U2 output is shown in Figure 7 and is divided into the following steps:

1. Forward charging: u_1_ is connected to −2.5 V and u_2_ to ground and charged for a fixed time to ensure voltage output of the integrator circuit is positive.
2. Integrate −1 mV: u_1_ is connected to −1 mV, and u_2_ is connected to the output of the integrator circuit U2. The diode in the figure turns ON and communicates with the microprocessor.
3. The gear resistor is connected to charge the integrator circuit. u_1_ is connected to the output of the signal acquisition unit when detecting the voltage at both ends of the gear resistance, u_2_ is grounded, *u_R_* is the voltage value at both ends of the gear resistance, and the timer is opened. After *t*_1_, the voltage output of the integrator circuit is
  (1) $$u_{g}=-\frac{1}{R_{5}C_{1}}u_{R}t_{1}+(-1mV).$$
4. The integrator circuit performs the discharge process. u_1_ is grounded, u_2_ is connected to −2.5 V, and the microprocessor is set to trigger at the falling edge. −2.5 V passes through U1 and enters the reverse input of U2. The output voltage of U2 begins to rise along a straight line with a slope of 2.5/*R*_5_*C*_1_. When it increases to 0, a falling edge signal is generated. The microprocessor timer is turned OFF, and the discharge time *t*_3_ is recorded.
  (2) $$t_{3}=\frac{-u_{g}\times R_{5}C_{1}}{2.5}$$
5. Repeat steps (1) and (2).
6. The conductivity sensor is connected to charge the integrator circuit. u_1_ is connected to the output of the signal acquisition unit when detecting the voltage at both ends of the conductivity sensor, u_2_ is grounded, *u_RX_* is the voltage at both ends of the conductivity sensor, and the timer is opened. After *t*_1_, the voltage output of the integrator circuit is
  (3) $$u_{c}=-\frac{1}{R_{5}C_{1}}u_{RX}t_{1}+(-1mV).$$
7. The integrator circuit performs the discharge process. u_1_ is grounded, u_2_ is connected to −2.5 V, and the microprocessor is set to trigger at the falling edge. −2.5 V passes through U1 and enters the reverse input of U2. The output voltage of U2 begins to rise along a straight line with a slope of 2.5/*R*_5_*C*_1_. When it increases to 0, a falling edge signal is generated. The microprocessor timer is turned OFF, and the discharge time *t*_4_ is recorded.
  (4) $$t_{4}=\frac{-u_{c}\times R_{5}C_{1}}{2.5}$$
8. Repeat steps (1) and (2).
9. A discharge time *t*_2_ of −1 mV was measured. u_1_ is grounded and u_2_ connected to −2.5 V. Simultaneously, the timer is turned ON and the falling edge trigger is set. Further, −2.5 V passes through U1 and enters the reverse input terminal of U2. The output voltage of the integrator circuit starts from −1 mV and rises along a straight line with a slope of 2.5/*R*_5_*C*_1_. When it increases to 0, the diode is interrupted, the microprocessor pin receives the falling edge signal, and the timing is stopped. Subsequently, the discharge time *t*_2_ from −1 mV to 0 is recorded.

The equivalent resistance of the conductivity sensor is expressed as follows:

(5) $$R_{X}=\frac{t_{4}-t_{2}}{t_{3}-t_{2}}\times R,$$

where *R_X_* is the equivalent resistance of the measured liquid, *t*_2_ is the discharge time of −1 mV, *t*_3_ is the discharge time of the voltage at both ends of the gear resistance, *t*_4_ is the discharge time of the voltage at both ends of the conductivity sensor, and *R* is the value of the gear resistance.

### 3.3. Temperature Signal Acquisition Unit

**Figure 8:**
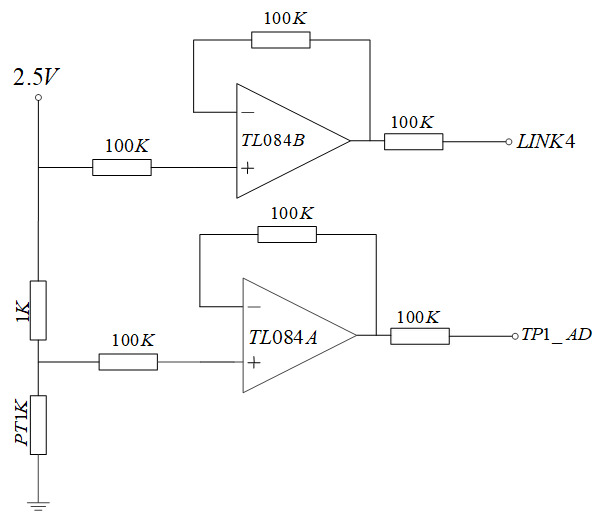
Circuit diagram of the temperature signal processing unit.

**Figure A1:**
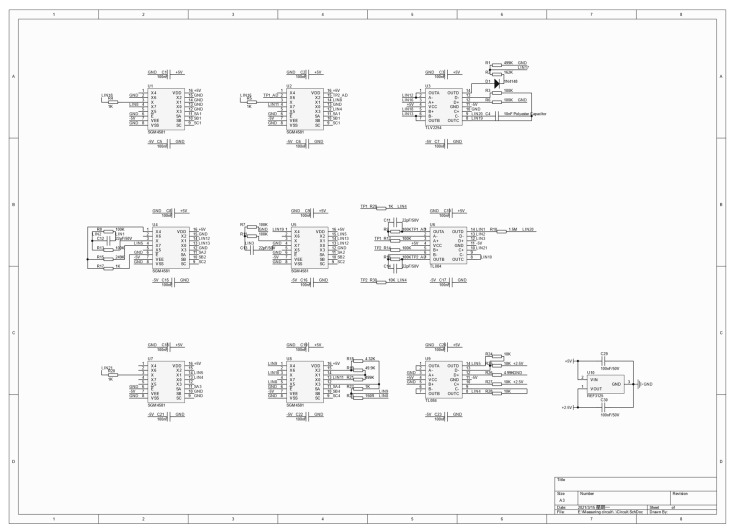
Circuit diagram of measuring circuit.

The temperature acquisition circuit designed in this study is shown in Figure 8. The 2.5 V voltage is connected to the temperature sensor through a 1 KΩ resistance sampling resistor. The operational amplifier TL084 and 100 KΩ resistance constitute a voltage follower in the same direction. The resistance values of temperature sensor PT1K were obtained by collecting the voltage at both ends of the sampling resistor and the voltage at both ends of the temperature sensor, and then the temperature was calculated (For other hardware circuits, please refer to our hardware circuit diagram Figure A1).

## 4. Method of Gear Control Based on Fuzzy Control

The formula of conductivity is as follows:

(6) $$e_{t}=Q/R_{X},$$

where *e_t_* is the conductivity, *R_X_* is the resistance of the measured solution, and *Q* is the electrode constant, which can be defined by the following formula:

(7) Q=L/A,

where *A* is the effective plate area of the measuring electrode and *L* is the distance between the two electrodes. *Q* is determined by the conductivity sensor used. 

The temperature compensation formula of conductivity is as follows:

(8) $$e_{t}=e[1+\alpha (t-t_{cal})],$$

where *e_t_* is the conductivity at a certain temperature, *e* is the conductivity at the standard temperature, which we can measure, *α* is the solution temperature coefficient at the standard temperature, we can query the data to determine its value, *t* is the measured temperature, we can measure, *t_cal_* is the standard temperature, here we take 25 °C.

Through simultaneous Formulas (6) and (8), we can get the expression of the equivalent resistance of the measured solution as follows:

(9) $$R_{X}=\frac{Q}{e[1+\alpha (t-t_{cal})]}.$$

**Figure 9:**
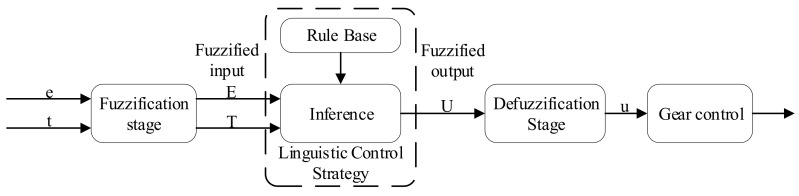
Fuzzy control structure.

Because our gear resistance and conductivity sensor are connected in series, when the difference between the equivalent resistance of the measured solution and the gear resistance is too large, the measurement error will occur. We hope that the resistance value of the gear resistance *R* and the equivalent resistance value of the measured solution *R_X_* are similar, which requires that we must ensure that the gear resistance should be replaced with different resistance values in real time, therefore, we control the value of the gear resistance according to the measured conductivity *e* and temperature *t*. Fuzzy control has the advantages of a fast response speed and high stability [31,32,33]. In this study, a fuzzy control algorithm is used to realize gear switching to maintain the gear resistance and the measured liquid resistance at a similar level. Figure 9 shows the structure of the implemented fuzzy logic algorithm.

**Figure 10:**
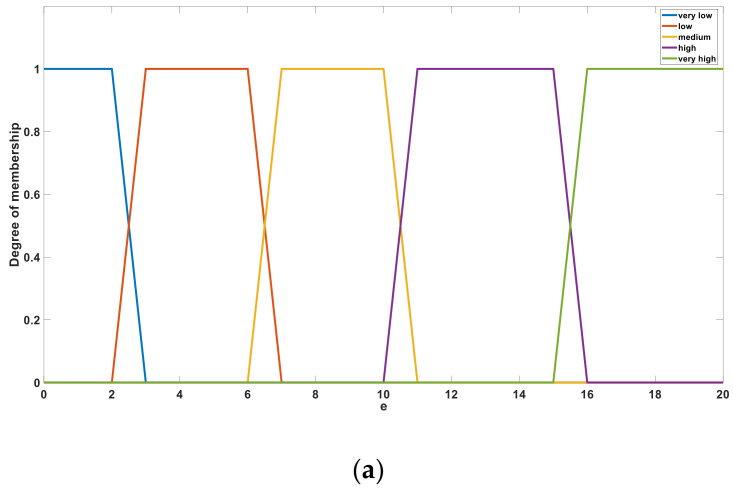

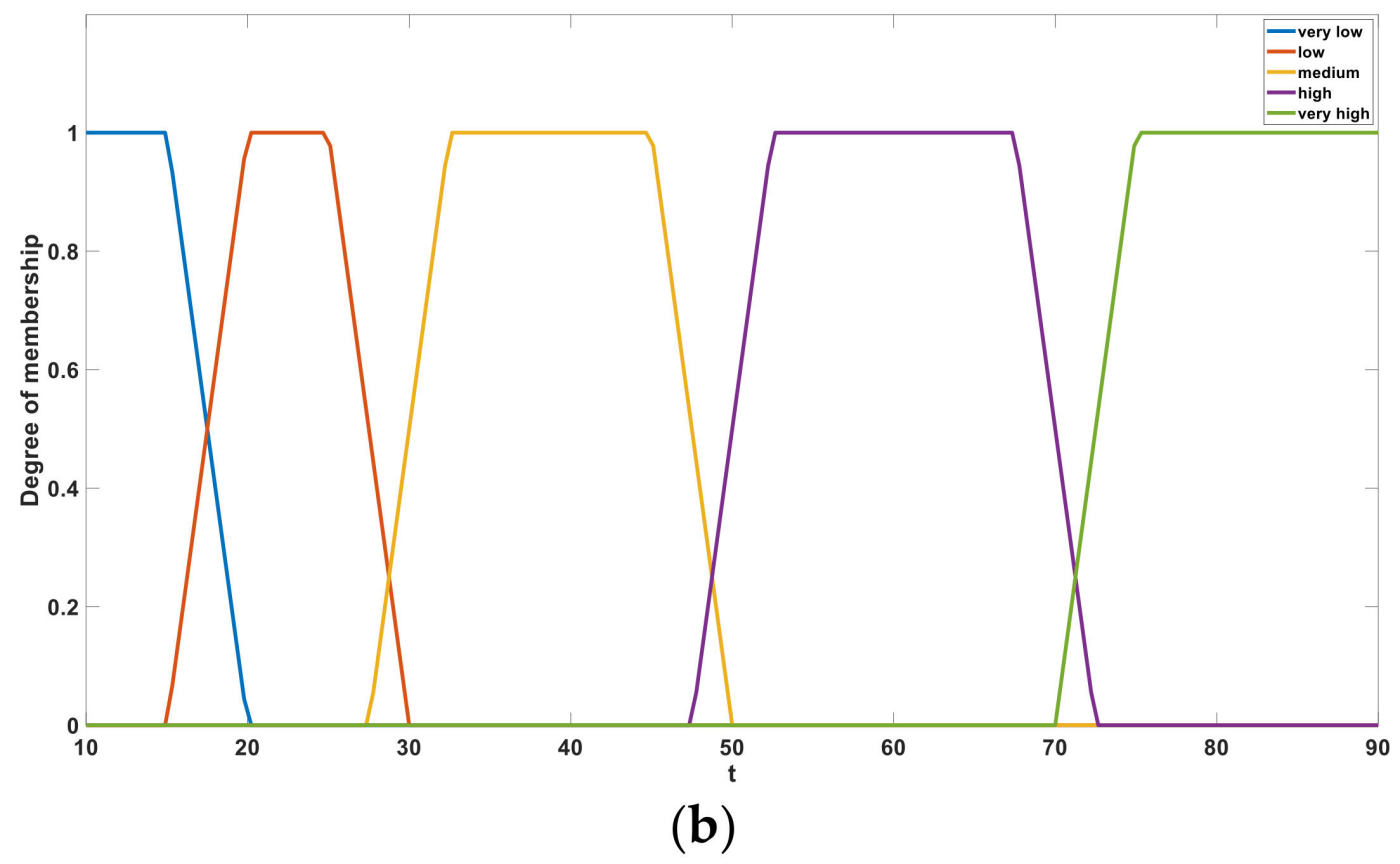
Membership functions of the input variable: (**a**) *e* and (**b**) *t*.

The algorithm considers the measured conductivity *e* and temperature *t* as input variables. In the fuzzification stage, five membership functions are defined for each input variable of the fuzzy algorithm, and a trapezoid function was selected among several commonly used membership functions [34]. Figure 10a,b shows the membership sets of *e* and *t*, respectively. After fuzzification, the variables *e* and *t* are defined using the five language descriptions of “very low”, “low”, “medium”, “high”, and “very high”. These language descriptions behave as linear correlation functions in a certain interval and act as fuzzy subsets in a single bit interval [0, 1].

The Mamdani fuzzy reasoning algorithm was used, and the reasoning rules were set based on experimental knowledge and calculations. A total of 25 rules are listed in Table 1.

**Figure 11:**
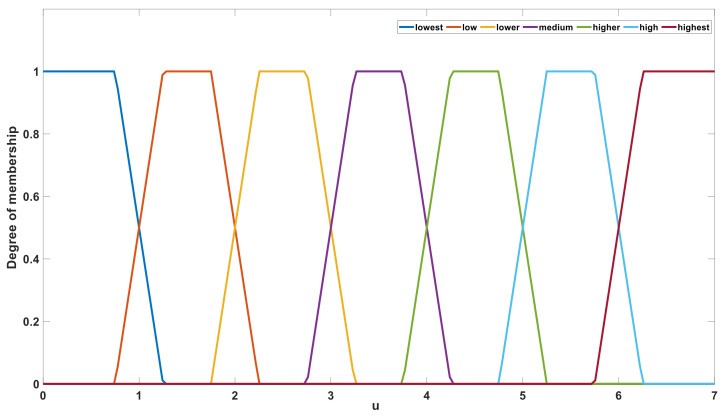
Membership function of the output variable *u*.

After fuzzy reasoning based on fuzzy rules, the output value *u* was defined using the seven language descriptions of “lowest”, “low”, “lower”, “medium”, “higher”, “high”, and “highest”. Figure 11 shows the membership set of output value *u*. In the defuzzification stage, the value of *u* is calculated according to the language description and membership function. The barycenter method was selected from among several calculation methods [35,36]. After defuzzification stage, the range of output *u* is [0, 7]. After an upward rounding operation, the value range of *u* is an integer within [1, 7]. The output value *u* is used to control the gear of the gear resistance. Corresponding to the change of *u*, the measuring system will use different gear resistance to connect to the circuit, which ensure that the gear resistance used matches the equivalent resistance of the solution to be measure. This ensured that the error was small. If the output value *u* is 1, the system will connect the minimum gear resistance to the measurement circuit. If the output value *u* is 7, the system will connect the maximum gear resistance to the measurement circuit. The hardware circuit of gear control is the U8 chip and its peripheral circuit in Figure A1.

By using the method of gear control based on fuzzy control, according to the measured temperature and conductivity value, the appropriate gear resistance was adopted. The value of the gear resistance connected to the integration circuit is close to the resistance value of the measured solution, which can reduce the measurement error caused by the large difference between the two resistance values.

## 5. System Object

**Figure 12:**
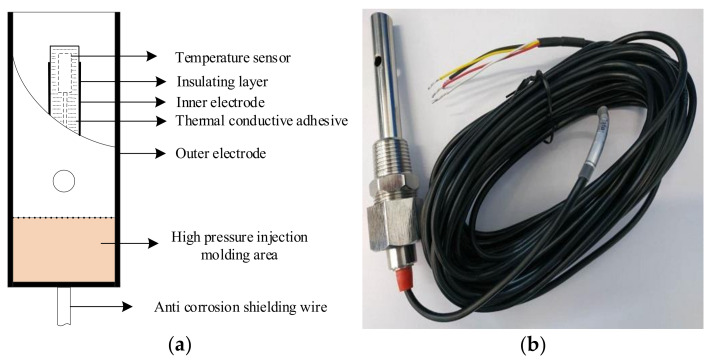
(**a**) Internal structure and (**b**) physical image of the sensor.

The internal structure of the two-electrode conductivity sensor designed in this study is shown in Figure 12. The inner electrode is connected to an excitation signal. To control the electrode constant, a part of the sensor was wrapped with an insulating layer. The outer electrode was the receiving electrode, and two symmetrical circular water outlets are available on the outer electrode to allow the measured solution to flow through. The temperature sensor PT1000 was fixed to the inner electrode using a thermally conductive adhesive. The red and black wires are the signal wires of the temperature sensor, the white wire is connected to the inner electrode of the conductivity sensor, and the yellow wire is connected to the outer electrode of the conductivity sensor. The electrode constant of our conductivity sensor is 10.06.

**Figure 13:**
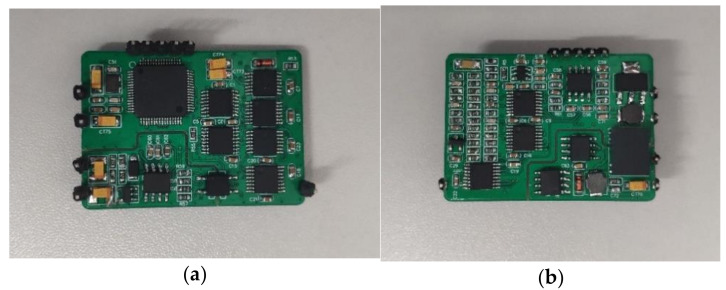
Hardware circuit photos: (**a**) front and (**b**) flank.

The hardware circuit used in this study is shown in Figure 13. There are 10 interfaces in the circuit, among which four interfaces are used to connect the conductivity sensor and the temperature sensor PT1000 embedded in the conductivity sensor, three interfaces were used for RS485 communication, and two interfaces were used for the system power supply. The lower right interface in Figure 13a is the physical support and does not need to be connected when the circuit is running. The volume of the hardware circuit is very small, that is, 30 mm × 42 mm × 13 mm. This volume parameter includes the pins to be soldered for experimental wiring. The height of the hardware circuit can be further reduced when it is packaged and used, and it can be easily embedded into other instruments or systems.

**Figure 14:**
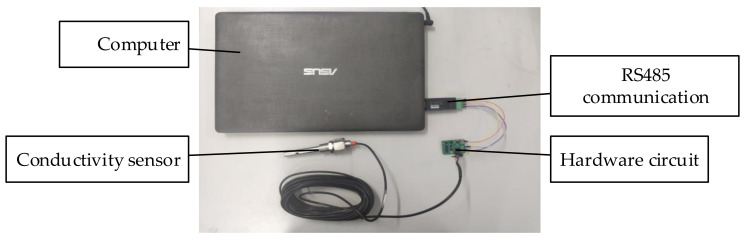
Image of the unsealed system.

An image of the unsealed system is shown in Figure 14. The system is composed of a conductivity sensor, hardware circuit, and upper computer. In terms of communication mode, RS485 communication was used to ensure a sufficient communication distance, and the working frequency of the system was set to 5 kHz.

## 6. System Experiment

This section introduces the experiment of the system, including the determination of excitation signal frequency, conductivity measurement calibration, model establishment, precision test, contrast experiment, repeatability test, and heat resistance test. To determine the excitation signal frequency, the waveforms of the conductivity sensor under different square wave signal frequencies were compared. Curve fitting and linear regression reduced the conductivity measurement errors in the conductivity-calibration test. The relationships among temperature, conductivity, and salinity were established in the model experiment, and the salinity of the solution was output according to the mathematical model of the relationship among the three. The accuracy and repeatability of the system were tested. In the contrast experiment, the output of the system based on fuzzy control was compared with that of the system without fuzzy control. In addition, a heat resistance test was conducted to determine whether the system could operate stably in a high-temperature environment.

### 6.1. Determination of the Excitation Signal Frequency

**Figure 15:**
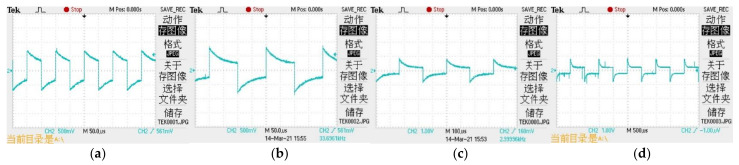
Waveforms at both ends of conductivity sensor under different frequency excitation signals: (**a**) 10 kHZ, (**b**) 5 kHZ, (**c**) 3 kHZ, and (**d**) 1 kHZ.

Before beginning all the experiments, the frequency of the excitation signal should be first determined. Square wave signals of 10, 5, 3, and 1 kHz were used as the excitation signals of the system. The waveforms at both ends of the conductivity sensor are shown in Figure 15.

It can be observed from the experimental results that the frequency of the excitation source signal at 10, 5, and 3 kHz is greatly affected by the capacitance effect; hence, an accurate measurement value is difficult to obtain. When the excitation source signal is 1 kHz, a time period is less affected by the polarization effect and capacitance effect exists, which is convenient for measurement. This experimental result is consistent with the conclusions of Hubálek [30]. Concomitantly, owing to the unacceptable measurement error when measuring high conductivity under a low-frequency excitation signal, the excitation signal frequency of this system is 1 kHz.

### 6.2. Conductivity Measurement Calibration

Second, the conductivity of the system is calibrated. Ten groups of potassium chloride (KCl) standard solutions with different conductivities were measured five times at 25 °C. The average value was recorded, and the errors were calculated. The experimental data are listed in Table 2.

According to the experimental data, the measured conductivity was significantly smaller than the actual conductivity of the solution. This is due to the impedance of the measurement system, which is composed of three parts. The first part is the resistance of the solution to be measured and the electrochemical double-layer capacitance caused by the capacitance effect; the second part is the capacitance generated by the conductivity sensor, and the third part is the capacitive reactance produced by various parasitic capacitors in the wiring and electronic devices.

In this study, a fitting calibration method is used to reduce the error. The measured conductivity value was fitted, and the following fitting formula was obtained:

(10) $$F(x)=1.006x+0.1472,R^{2}=1$$

**Figure 16:**
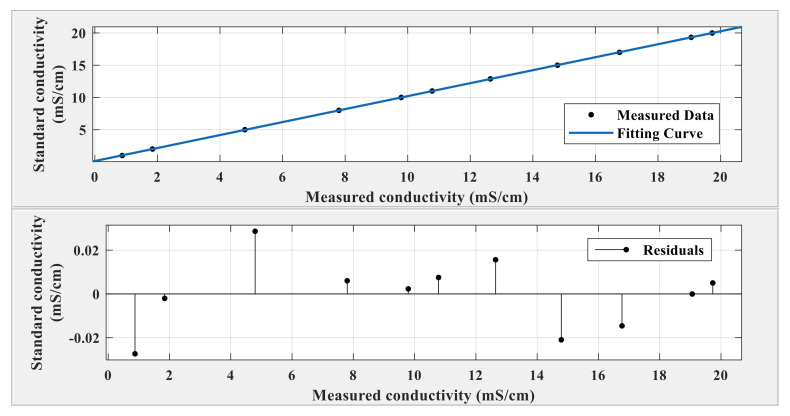
Fitting curve and residual error after fitting.

In the above formula, *x* is the measured conductivity value and *F*(*x*) is the standard conductivity value. Figure 16 demonstrates the fitting results and the residual error after fitting.

As shown in Figure 16, the residual error after fitting was not more than 0.03, and the conductivity measurement error of the system was not more than 0.03 mS/cm after theoretical calibration.

### 6.3. Model Establishment

To determine the relationship between conductivity, salinity, and temperature, a mathematical model was established to measure the relationship between salinity and conductivity when the temperature was constant and between temperature and conductivity when the salinity was constant. Prior to the experiment, the salinity of the water injection solution of a certain oil well in Daqing was found to be between 4 g/L and 6 g/L, as measured by the instrument DDSJ-308A. To ensure the system could meet the required measurement, 10 different concentrations of KCl sample solutions with mineralization degrees ranging from 1 g/L to 10 g/L were configured and heated in the incubator. The recorded conductivity values were 20, 30, 40, 50, 60, 70, 80, and 90 °C. Each sample was measured five times at each temperature, and the average value was obtained. The results are shown in Table 3.

**Figure 17:**
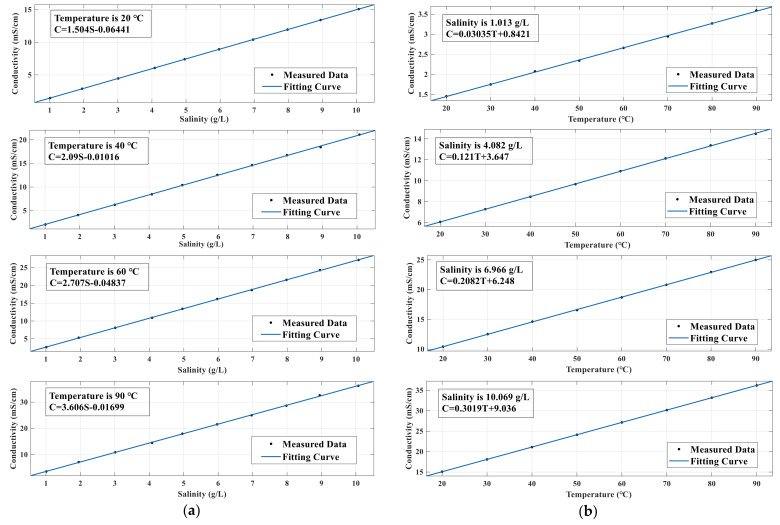
All other conditions held constant: the relationship between different salinity, temperature, and electrical conductivity. (**a**) Relationship between different salinity conditions and electrical conductivity at 20, 40, 60, and 90 °C. (**b**) Relationship between temperature and conductivity under the salinity conditions of 1.013 g/L, 4.082 g/L, 6.966 g/L, and 10.069 g/L.

Using the data in Table 3, the broken line graph of the relationship between conductivity and salinity at different temperatures is shown in Figure 17a, and the relationship between temperature and conductivity at different salinity conditions is shown in Figure 17b. When the temperature was constant, the conductivity had a linear relationship with salinity, and the slope increased with an increase in temperature. When the salinity was constant, the conductivity had a linear relationship with temperature, and the slope increased with increasing salinity.

**Figure 18:**
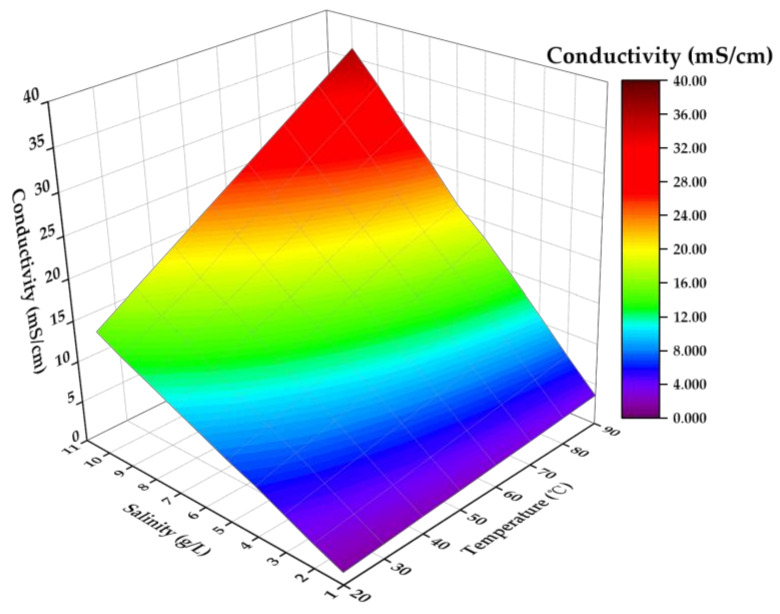
Relationship between salinity, temperature, and conductivity.

Using the above data, the relationship between conductivity, salinity, and temperature was drawn, as shown in Figure 18, and the relationships among the three were fitted.

(11) $$S=\frac{C+0.03412+0.001022T-0.0001384T^{2}}{0.8985+0.03011T},$$

where *C* and *T* denote the conductivity and temperature of the solution, respectively. After the conductivity and temperature of the solution were measured by the system, the salinity value *S* of the solution was output according to the above relationship.

### 6.4. Accuracy Test

Two parts each of 0.250, 0.500, 1.250, 2.000, and 2.500 g KCl particles were weighed using a Shanghai Anrui company AR1140 electronic balance, one for the heating test and the other for backup. Moreover, 10 parts of 250 mL of distilled water were measured in a long beaker, and the above 10 parts of KCl particles were dissolved in distilled water and mixed with a glass rod. After preparation, five solutions were used for the thermal test, and five duplicates were set aside. The beaker with the solution was placed on a temperature-controlled magnetic stirrer (SCZL-2 type, Yuhua Company, Guangzhou, China) for heating. Simultaneously, the conductivity sensor was placed in a beaker, and the data were recorded every 5 °C during heating. The mineralization data output of the system is listed in Table 4.

The experimental data indicated that the maximum error was 0.06 g/L in the range of 1–10 g/L, and the accuracy of the system was 0.6%. According to the Chinese standard “GBT 13283-2008 industrial process measurement and control instrumentation accuracy class” for instrument accuracy, the accuracy of this measurement system met the requirements of a class 1.0 instrument. The error was caused by two reasons: first, the inevitable factors in the conductivity measurement as described above and second, the fitting residual error of the relationship model of temperature, conductivity, and salinity. The fitting residual error can be slightly reduced by high-order fitting. However, the benefit is insignificant, and it would increase the calculation to be processed by the microprocessor; thus, it was not used.

### 6.5. Contrast Experiment

The Shanghai Anrui company AR1140 electronic balance was used to weigh 0.250, 0.500, 0.750, 1.000, 1.250, 1.500, 1.750, 2.000, 2.250, and 2.500 g KCl particles, respectively, one for comparative experiment and the other for backup. Further, 10 parts of 250 mL of distilled water were measured in a long beaker, and the above 10 parts of KCl particles were dissolved in the distilled water and mixed with a glass rod. The measurement was carried out in the case of using the algorithm of changing gears based on fuzzy control and in the case of not using the algorithm respectively, five times in each case, and the average value was obtained. The salinity data output by the system under the two conditions are shown in Table 5.

Compared with the case without changing the gear, the error of the system is clearly reduced, and the output is more stable. It can be observed that the gear change method proposed in this study can effectively reduce the measurement error.

### 6.6. System Repeatability Test

Five KCl solutions with different salinity conditions were randomly proportioned, and each solution was measured 10 times. The experimental data are presented in Table 6.

According to the repeatability experiment, the maximum repeatability error was 0.02 g/L, and the uncertainty was 0.2%. The repeatability of the system was found to be appropriate.

### 6.7. System Heat Resistance Test

**Figure 19:**
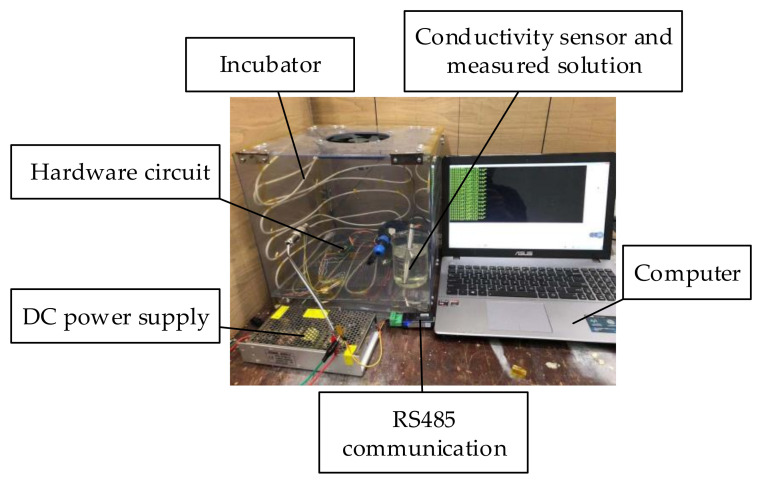
Experimental diagram of high temperature resistance of system hardware.

**Figure 20:**
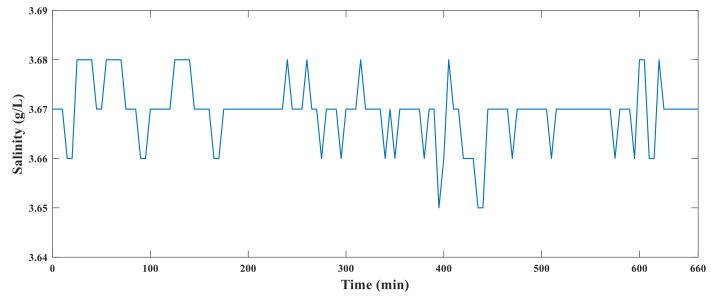
Results of high temperature test.

The conductivity sensor, hardware circuit, and solution to be measured were placed in an incubator. The temperature in the incubator was gradually increased from 20 °C to 85 °C. After 10 h of heating, the temperature of the incubator was gradually reduced to 20 °C. During this process, the system data were recorded every 10 min. The experimental diagram of the system is shown in Figure 19, and the results of the high-temperature resistance test are shown in Figure 20.

Figure 20 shows that the system runs stably and the measured data did not fluctuate substantially, indicating that the hardware of the system operated normally at 85 °C. Because the target ambient temperature of the sensor is 60–80 °C and the probability of working in an environment of 85 °C is small, we believe that the hardware part of the measurement system will run successfully in the oilfield.

## 7. Conclusions

In this study, a real-time mineralization detection system based on fuzzy control, composed of a conductivity sensor, hardware circuit, and upper computer, was proposed and designed. Based on the analysis of various conductivity acquisition methods, a series of methods have been proposed to reduce system errors. These include using a bipolar square wave as an excitation source and integrator circuits as the core of the signal acquisition circuit and introducing fuzzy control into the shift of sampling resistance to reduce the system error. By establishing a model of temperature, conductivity, and salinity, the salinity of the measured liquid can be calculated according to the temperature and conductivity measured by the system.

The experimental results show that the accuracy of the system was 0.6%, and the uncertainty was 0.2% in the range of 1–10 g/L. The system exhibited suitable repeatability, and the hardware circuit ran stably in a high-temperature environment. It can successfully detect the real-time salinity of a water injection solution in oil–water injection engineering.

## Figures and Tables

**Table 1:** Fuzzy rule table for *u.*

		*e*				
		Very Low	Low	Medium	High	Very High
***t***	**Very low**	highest	highest	high	higher	medium
	**Low**	highest	high	higher	medium	lower
	**Medium**	high	higher	medium	lower	low
	**High**	higher	medium	lower	low	lowest
	**Very high**	medium	lower	low	lowest	lowest

**Table 2:** Conductivity calibration data.

Standard Conductivity (mS/cm)	Measured Conductivity (mS/cm)	Error (mS/cm)
1.00	0.875	0.125
2.00	1.844	0.156
5.00	4.796	0.204
8.00	7.801	0.199
10.00	9.793	0.207
11.00	10.782	0.218
12.88	12.643	0.237
15.00	14.787	0.213
17.00	16.769	0.231
19.32	19.061	0.259
20.00	19.732	0.268

**Table 3:** Conductivity of solutions with different salinity conditions at different temperatures (unit: mS/cm).

Temperature (°C)	Salinity (g/L)									
	1.013	1.953	3.006	4.082	4.963	5.974	6.966	7.957	8.950	10.069
20	1.454	2.878	4.458	6.070	7.400	8.921	10.412	11.939	13.411	15.083
30	1.750	3.485	5.357	7.283	8.906	10.739	12.520	14.342	16.127	18.093
40	2.075	4.071	6.242	8.469	10.415	12.553	14.624	16.740	18.454	21.098
50	2.344	4.694	7.171	9.674	11.937	14.342	16.570	19.154	21.543	24.125
60	2.660	5.294	8.092	10.913	13.419	16.171	18.703	21.565	24.284	27.152
70	2.946	5.905	8.981	12.133	14.952	17.992	20.822	24.019	26.986	30.163
80	3.270	6.523	9.915	13.369	16.473	19.781	22.947	26.453	29.713	33.199
90	3.592	7.146	10.877	14.492	17.954	21.519	24.985	28.642	32.571	36.201

**Table 4:** Accuracy of the test data.

Salinity of Solution (g/L)	Minimum Output from 20 °C to 90 °C (g/L)	Maximum Output from 20 °C to 90 °C (g/L)	Maximum Error (g/L)
1.00	0.97	1.02	0.03
2.00	1.98	2.04	0.04
5.00	4.95	5.03	0.05
8.00	7.95	8.06	0.06
10.00	9.94	10.04	0.06

**Table 5:** Comparison of the experimental data.

Salinity of Solution/(g/L)	Measured Values with the Change Gear Method/(g/L)	Error/(g/L)	Measured Values without the Change Gear Method/(g/L)	Error/(g/L)
1.00	1.00	0.00	1.02	0.02
2.00	1.99	0.01	2.05	0.05
3.00	3.01	0.01	3.04	0.04
4.00	3.99	0.01	4.09	0.09
5.00	4.98	0.02	5.11	0.11
6.00	6.01	0.01	6.07	0.07
7.00	6.97	0.03	7.15	0.15
8.00	7.97	0.03	8.08	0.08
9.00	8.96	0.04	9.17	0.17
10.00	9.96	0.04	10.23	0.23

**Table 6:** Repeatability of the test data.

Solution Number	Measured Salinity (g/L)										Maximum Error (g/L)
1	2.55	2.53	2.54	2.54	2.55	2.55	2.54	2.53	2.53	2.54	0.02
2	3.16	3.14	3.16	3.16	3.16	3.16	3.15	3.14	3.15	3.16	0.02
3	5.33	5.33	5.34	5.33	5.34	5.34	5.33	5.33	5.33	5.33	0.01
4	7.84	7.85	7.85	7.85	7.85	7.84	7.84	7.85	7.84	7.85	0.01
5	9.41	9.39	9.39	9.39	9.39	9.40	9.39	9.40	9.41	9.39	0.02

## Data Availability

In our article, all the data are disclosed and explained in different parts of the article.
